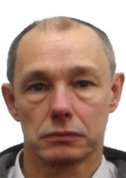# Advances in Organic Anode Materials for Na‐/K‐Ion Rechargeable Batteries

**DOI:** 10.1002/cssc.202001334

**Published:** 2020-08-14

**Authors:** Aamod V. Desai, Russell E. Morris, A. Robert Armstrong

**Affiliations:** ^1^ EastChem School of Chemistry University of St. Andrews North Haugh St. Andrews KY16 9ST United Kingdom; ^2^ The Faraday Institution Quad One Harwell Science and Innovation Campus Didcot OX11 0RA United Kingdom; ^3^ Department of Physical and Macromolecular Chemistry, Faculty of Science Charles University Hlavova 8 128 43 Prague 2 Czech Republic

**Keywords:** sodium-ion, potassium-ion, battery, organic electrode, anode

## Abstract

Electrochemical energy storage (EES) devices are gaining ever greater prominence in the quest for global energy security. With increasing applications and widening scope, rechargeable battery technology is gradually finding avenues for more abundant and sustainable systems such as Na‐ion (NIB) and K‐ion batteries (KIB). Development of suitable electrode materials lies at the core of this transition. Organic redox‐active molecules are attractive candidates as negative electrode materials owing to their low redox potentials and the fact that they can be obtained from biomass. Also, the rich structural diversity allows integration into several solid‐state polymeric materials. Research in this domain is increasingly focused on deploying molecular engineering to address specific electrochemical limitations that hamper competition with rival materials. This Minireview aims to summarize the advances in both the electrochemical properties and the materials development of organic anode materials.

## Graphical abstract

**Sustainable electrodes**: Sodium‐ion and potassium‐ion batteries are likely to contribute significantly in the upcoming years to global energy security. Sustainable electrode materials must be developed to enable this growth. Organic molecules are at the core of this technology and materials offering low‐voltage redox are improving. This Minireview tracks the progress and remaining challenges in this domain.

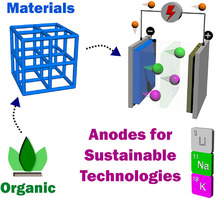

## 1 Introduction

Global energy demands are likely to see a remarkable upsurge over the next couple of decades.^1^ The projections vary depending upon several practical factors, necessitating wider deviation windows.^2^ Apart from achieving global energy security, the depletion of fossil fuels and the pressing concern to mitigate climate change have emphasized the transition to low‐carbon energy.^3^ Renewable sources are anticipated to play a crucial role in addressing most of these challenges.^4^ The majority of renewable sources are intermittent in nature,^5^ which has prompted extraordinary research interest in electrochemical energy storage (EES) technologies to tap renewable energy effectively.^6^ The added incentive for the development of EES is the push for electricity as the preferred energy carrier, owing to higher efficiency and cleaner mode of energy consumption.^7^ The contribution of renewable energy is growing,^8^ and there remains substantial untapped potential for EES technologies.^9^

The widespread commercialization and successful applications of rechargeable Li‐ion batteries (LIB) have demonstrated the opportunities that exist for battery systems.^10^ The higher conversion efficiency, energy density, lifespan and cost competitiveness of rechargeable battery technology has further driven its exploration.^11^ To meet growing demand, a major challenge is the uninterrupted supply of lithium, owing to low natural abundance^12^ and uneven geographical distribution.^13^ This has prompted intense research interest in developing similar technologies based on more abundant elements. The subsequent two elements in Group 1 exhibit similar physicochemical characteristics to those of lithium, which has resulted in the promising development of Na‐ion (NIB)^14^ and K‐ion batteries (KIB).^15^ The features driving this progress are abundance, costs of raw materials^16^ and early studies showing promising life cycle assessments.^17^ Although the transition of electrode materials from LIB to NIB/KIB would seem straightforward, in practice significant differences in chemical properties and performance are observed.^18^ For instance, graphite is the popular choice of anode for LIBs, but the intercalation of sodium is disfavored.^19^ In fact, while the concept of NIBs came to the fore in parallel with the advent of LIBs, it fell behind owing to lack of a suitable anode material.^20^ Another negative electrode material which has caught attention in LIBs is silicon, but this does not work for sodium insertion owing to sluggish kinetics.^21^ Similarly, inorganic electrode materials undergo remarkable volume change during potassium‐ion insertion.^22^ These differences stem from the dissimilar physical and electrochemical properties of the three elements/ions (Table 1).^23^

**Table 1:** Comparison of physical properties of lithium, sodium and potassium and corresponding cations.

Element (Ion)	Abundance in earth's crust [wt %]	*E*°^[a]^ [V_SHE_]	Shannon's ionic radius [Å]	Stokes radius^[b]^ [Å]	Atomic mass
lithium (Li^+^)	0.002	−3.04	0.76	4.8	6.94
sodium (Na^+^)	2.4	−2.71	1.02	4.6	22.99
potassium (K^+^)	2.1	−2.93	1.38	3.6	39.10

[a] A^+^_aq._/A, V_SHE_: V versus the standard hydrogen electrode. [b] In propylene carbonate.

Given these considerable differences, development of suitable battery materials for NIBs/KIBs has been recognized as the key step for their progress.^24^ In pursuit of realizing this target, the broader objectives such as green, sustainable development and ambitious demands of future applications also need to be incorporated.^25^ In this regard, significant research attention has been devoted to improvement of cathode materials. On the other hand, the materials for the anode are relatively less explored. Anode materials for emerging battery technologies can be broadly classified into carbonaceous, organic materials, alloys and metal salts such as oxides, sulfides and phosphides.^26^ Among these, organic materials, in principle, can deliver truly sustainable battery components as organic molecules can be derived from biomass. Also, these molecules can be integrated into different types of materials, which offers a wide range to explore and modulate features for practical applications. Although there has been remarkable progress in recent years, there remain several factors which need to be addressed for this class of materials to compete for practical implementation.

NIB and KIB are the upcoming EES technologies and in recent years, several reviews have been published covering different aspects of NIBs and KIBs.^27^ We believe that given the rapidly evolving nature of research in this field and concurrent growing scope of applications, focused reviews can effectively assist the overall progress of the field. The field of organic electrode materials for metal‐ion batteries is seeking greater prominence in recent years and more emphasis is being directed to finetuning molecular properties.27e, 27g, 28 Given the diversity of materials and redox behavior, and their growing applicability, specific classification of materials can complement active research. In this minireview we present a progress of organic materials for negative electrodes for both NIBs and KIBs. The different material classes, working principles, advantages and electrochemical features or limitations are outlined, followed by specific examples. In conclusion, the future challenges and scope for these materials is discussed.

## 2 General Considerations for Organic Anode Materials

### 2.1 Organic anodes – working principles, types and advantages

**Figure 1:**
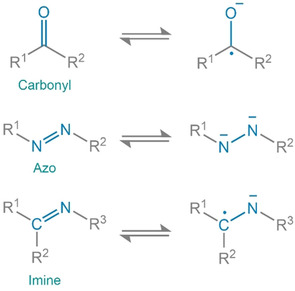
Redox states for organic moieties typically employed as anodes in secondary batteries.

The electrochemical function of organic molecules is based on the change in charge state of redox active moieties. The polarized state is utilized for interaction with the mobile ion. Depending upon the potential of this activity, the compounds can either be suited for use as positive or negative electrode. Concerning the change of charge‐states, organic systems can be classified as n‐type, p‐type or bipolar. Compounds which acquire a negative charge from the initial neutral state during redox reaction and revert to their original state by oxidation are categorized as n‐type. Likewise, p‐type systems obtain a net positive charge from the initial neutral state in the redox process. Bipolar systems can access both positive and negative states from the neutral state. In terms of molecular character, several active groups such as carboxylate, imide, anhydride, quinone, ketone, azo, imine and pteridine have been found to show redox activity.^28^ For anodes, three major functional groups, carboxylate, imine and azo, are reported to be well suited ‐ having low redox potential (Figure 1). These redox active moieties are integrated into different types of molecules which are deployed as solid‐state electrode materials.

**Scheme 1:**
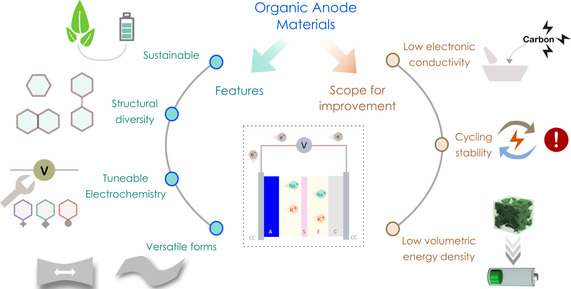
Illustration showing different aspects of organic anode materials. (CC – current collector, A – anode, S – separator, E – electrolyte, C – cathode).

Organic compounds are beneficial as electrode materials on several counts (Scheme 1). The ability to synthesize a myriad of organic molecules, delivers immense flexibility for exploration. The precise knowledge of the molecular arrangements provides a handle to tune structural features or modulate properties according to the demands of the desired application. Additionally, as specific active sites are involved in the redox process, irrespective of the backbone, the chemistry can be extended to several solid‐state materials.

Another major advantage of organic materials lies in meeting the goal of greener battery components. Typically, organic molecules are composed of elements (C, H, O, N, S) which are obtained from biomass,^29^ which eliminate residues from toxic elements. In recent years there has been a strong push to convert biomass into useful chemicals, particularly driven by the interest in reducing dependence on petroleum sources.^30^ Early studies suggest this approach is indeed feasible and competitive, and wider application can contribute cooperatively to the sustainability component of rechargeable battery technology.^31^ The synthesis of materials based on organic molecules involves relatively mild synthesis routes which are energetically economical. In addition to sustainability, organic materials are also cost effective. Few organic molecules have been tested as anodes for LIBs and other emerging battery technologies. A common feature is the low, but safer with respect to metal plating, working potentials. Metal deposition for sodium and potassium is of serious concern and hence the redox voltages of such systems are useful for practical applications. As organic systems carry out the redox activity by molecular conversion, a given compound, in principle, can be applied to storage of any similar ion (Li^+^, Na^+^, K^+^). This feature is more limited for inorganic materials, where analogous chemistry may lead to complications owing to high volume changes or result in poor kinetics.

Apart from material and redox features, organic compounds offer the scope for versatile workable forms, which are well suited for certain modern applications requiring stretchable or flexible battery design.^32^

### 2.2 Electrochemical aspects, limitations and optimisation

Any electrode material is evaluated based on several parameters such as capacity, cycling stability, energy density and rate capability. Both molecular characteristics and bulk properties are seen to influence one or more of these parameters.^33^ The effects of molecular properties on these factors are described with specific examples in Section 3.

The theoretical capacity of an active material is dependent on the number of electrons transferred and inversely proportional to the molecular weight. As organic materials show redox activity based on specific active sites, there is a greater proportion of inactive mass which invariably reduces the capacity of the electrode. Thus, increasing the density of active sites in any redox active organic molecule is necessary to improve the capacity. Although the molecular properties can improve theoretical capacity, several bulk properties of the material such as particle size and crystallinity are seen to influence the practical capacities.

Cycling stability and Coulombic efficiency are vital parameters for any electrode material to be considered for practical implementation. Typically, a polar organic electrolyte is employed in rechargeable batteries, making organic electrodes susceptible to dissolution or undesired side reactions. One approach to overcome solubility and improve electrode stability is to use polymeric solids instead of small molecules. Also, for anodes operating at lower voltages, the formation of a solid electrolyte interphase (SEI) layer leads to lower Coulombic efficiencies at early cycles. Such low initial efficiencies have been listed as major bottlenecks towards practical implementation.^34^ Cycling performance is also affected by large volume changes in the active material or transformation into an inactive phase. For bigger ions (Na^+^ or K^+^), some materials are seen to suffer from ion trapping, leading to much lower capacities. Porous materials have been proposed to address these challenges by virtue of possessing space to accommodate charges and provide a path for smoother ion diffusion. However, this is done at the cost of another crucial parameter – volumetric energy density.

Energy density is a vital parameter for evaluating battery performance and has a contribution to the cost and environmental impact of an electrode material.^35^ Energy density can be tuned by using a cathode operating at higher voltage, or an anode operating at lower voltage. However, for Na^+^ and K^+^ storage the safety concerns of metal plating are significantly higher, particularly with the high reactivity and low melting points of bare metals (97.7 °C and 63.5 °C respectively). In case of a given organic molecule, the working voltage drops with increasing ionic size of the interacting ion, owing to the weaker K−O and Na−O bond compared with Li−O.^36^ Also, the operating potential can be tuned by changing the electronic properties of organic molecules.

Another important criterion of performance is that of power density, which largely depends upon the intrinsic properties of the material. For high performance, both electron and ion transfer need to happen rapidly. This aspect is evaluated by rate capability, and strongly depends upon molecular character and packing structure. A major limitation of organic molecules, in general, is poor electronic conductivity (Scheme 1). Thus, organic electrodes require physical mixtures or composites with significant amounts of conducting carbon. This addition improves electronic response at the cost of increasing dead weight of the cell. Also, owing to larger size and mass, the capacity retention at higher rates is relatively lowered for Na^+^/K^+^ compared to Li^+^. However, for anode materials NIBs/KIBs offer an important advantage with the utilization of aluminum foil as the current collector. This has direct consequences on the cost of battery preparation and lower carbon footprint compared to that associated with the preparation of copper foils.^37^

## 3 Materials and Approaches

**Scheme 2:**
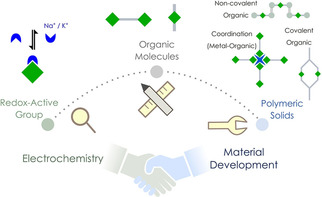
Schematic depiction of the progress from identifying redox‐active moiety to material development for further application.

Apart from understanding electrochemical features, molecular engineering and material optimization play a crucial role to achieve high‐performing systems. Research in organic systems typically ranges from identifying redox‐active moieties to forming stable molecules having higher density of active sites (Scheme 2). From an application perspective these molecules can then be assembled into coordination networks or covalently bonded polymers. The approaches of molecules to materials with an electrochemical context is discussed in this section.

### 3.1 Alkali metal coordination networks

Usually, alkali metal‐carboxylate networks have been proposed as efficient redox‐active organic materials for ion insertion. The highly ionic nature of the bond owing to the large electronegativity difference gives rise to a wide range of structures.^38^ These compounds incorporate the advantage of small organic units and long‐range ordering owing to networked structures. The different materials and approaches in alkali metal‐carboxylate compounds or organic molecules forming coordination compounds during cycling are presented below and representative examples listed in Table 2 and 3.

**Table 2:** Representative examples of sodium‐organic compounds used as negative electrodes, along with their compositions and brief performance upon cycling.^[a]^

Active compound	Composition	Voltage peak [V]		Capacity	Rate	Cycle	Ref.
	active/carbon/ binder	discharge	charge	[mAh g^−1^]	[mA g^−1^]	no.	
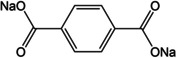	80 : 10 : 10 (Ketjenblack/PVDF)	0.29	0.56	192	0.1 C	50	[39]
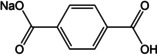	50 : 35 : 15 or 60 : 30 : 10 (Super P/sodium alginate)	0.34	0.50	244	20	50	[46]
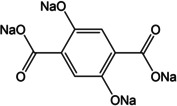	65 : 30 : 5 (Super P/PVDF)	0.15	0.40	184	19	100	[47]
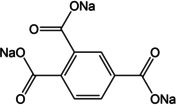	60 : 30 : 10 (carbon black/PVDF)	0.37	0.67	137	50	600	[50]
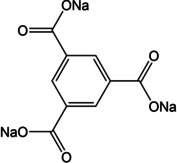	55 : 35 : 10 (Super P/PVDF)	0.42/0.09	0.22/0.57	75	10 C	1500	[51]
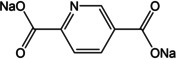	50 : 35 : 15 (Super P/PVDF)	0.50/0.38	0.72/0.85	225	0.1 C	100	[52]
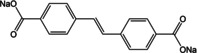	50 : 40 : 10 (Super P/CMC)	0.39	0.66	112	1000	400	[55]
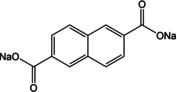	60 : 30 : 10 (Super P/CMC) or 65 : 10 : 15 : 10 (Super P/Ketjenblack/CMC)	0.38	0.50	170	100	200	[59]
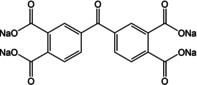	60 : 30 : 10 (Super P/sodium alginate)	0.8/0.3	1.4/1.7	158	50	100	[62]
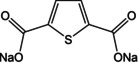	60 : 30 : 10 (Super P/sodium alginate)	0.75	1.05/2.00	334	50	200	[64]
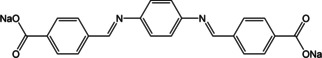	80 : 15/5 (Super C65/Ketjen black)	0.53/0.75/0.85	0.62/0.90/1.01	225	0.1 C	25	[65a]
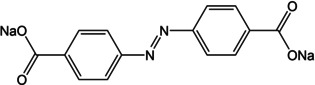	60 : 30 : 10 (carbon black/sodium alginate)	1.2/1.26	1.37/1.43	113	10 C	1000	[66]

[a] PVDF – poly(vinylidene fluoride), CMC – carboxymethyl cellulose.

**Table 3:** Representative examples of potassium‐organic compounds used as negative electrodes, along with their compositions and brief performance upon cycling.

Active compound	Composition	Voltage peak [V]		Capacity	Rate	Cycle	Reference
	active/carbon/binder	discharge	charge	[mAh g^−1^]	[mA g^−1^]	no.	
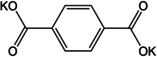	60 : 30 : 10 (Super P/PVDF)	0.44	0.71	194	1000	500	[69]
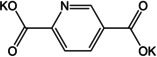	56 : 14 : 20 : 10 (Super P/acetylene black/PVDF)	0.6	0.8	176	0.2 C	100	[70]
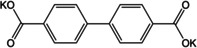	60 : 30 : 10 (Super P/CMC or PVDF)	0.35	0.97	120	20	100	[74]
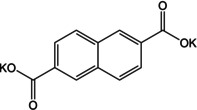	60 : 30 : 10 (Super P/PVDF)	0.22	0.89	116	50	200	[75]
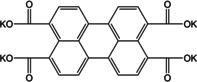	60 : 30 : 10 (Super P/PVDF)	0.92	1.48	74	50	300	[77]
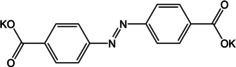	60 : 30 : 10 (Carbon black/Sodium alginate)	1.43/1.24	1.55/1.46	51	2 C	1000	[80]

#### 3.1.1 Sodium coordination compounds

**Figure 2:**
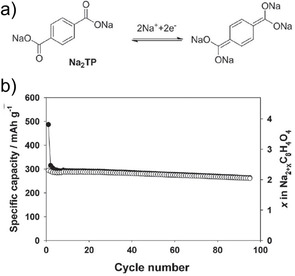
a) Chemical diagram showing molecular reorganization upon Na^+^ insertion. b) Cycling performance of Na_2_TP at current rate of 30 mAh g^−1^. Reproduced with permission.^40^ Copyright 2012, Wiley.

An early example came from Chen and co‐workers where disodium terephthalate (Na_2_TP) was shown as a useful anode material for NIBs.^39^ A low insertion potential of 0.29 V vs Na^+^/Na was reported with a reversible capacity of ∼250 mAh g^−1^. Additionally, the effect of atomic layer deposition (ALD) to reduce the effect of the SEI was examined by surface modification of the electrodes with Al_2_O_3_. The surface coating was found to enhance initial Coulombic efficiencies and provide improvement of rate capabilities. Almost simultaneously, Park et al. also reported the investigation of disodium terephthalate (Figure 2) and its substituents (−NH_2_, −NO_2_ and −Br).^40^ The capacity and working voltages were found to vary with the substitution, with the observation that electron donor groups decreased the discharge voltage, while electron withdrawing groups increased the redox potential relative to bare Na_2_TP.

Wang and co‐workers proposed an approach to improve the cycling stability of disodium terephthalate (Na_2_TP), by preparing a Na_2_TP@GE composite (GE stands for graphene).^41^ The composite exhibited significantly improved cycling performance, and 77.3 % retention of capacity after 500 cycles. Previously, Wang and co‐workers had reported the strategy of using graphene oxide to wrap the disodium salt of croconic acid.^42^ The improved cycling stability was ascribed to the retention of electrode structure and morphology, as the wrapping reduced the extent of pulverization caused by volume changes. Another aspect of material optimization in terms of particle size and morphology was reported by Wan et al.^43^ Nanosheets of Na_2_TP were synthesized and compared to the bulk material. The nanosheets were found have significantly superior performance over the bulk solid in terms of capacity and rate capability. A similar observation was reported by Tang and co‐workers, who carried out stepwise modulation of particle sizes.^44^ The material with smaller particle size was found to present significantly enhanced performance.

**Figure 3:**
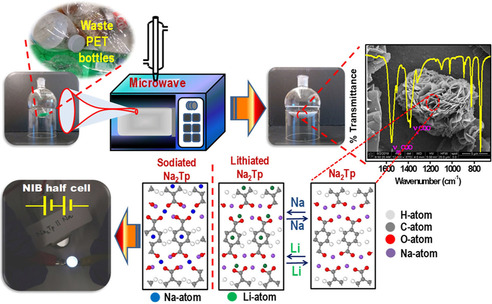
Flowchart of converting waste PET bottles to usable terephthalate for Na^+^/Li^+^ ion insertions. Reproduced with permission.^45^ Copyright 2020, American Chemical Society.

The major interest in the use of terephthalate is the possibility of large‐scale preparation from poly‐ethylene terephthalate (PET). Recently, Ghosh et al. reported a rapid preparation of terephthalate from waste PET and tested the material in a composite with Super P carbon (Figure 3).^45^ Microwave irradiation was used to decompose the PET polymer with NaOH and the subsequently obtained disodium terephthalate was cycled against both Li/Na ions.

Studies on electrode materials are usually reported on half cells. Amine and co‐workers reported the first instance of testing a full sodium‐ion cell using organic anodes of disodium and monosodium terephthalate.^46^ Additionally, cycling studies for a few other derivatives and bare linker were reported. The authors noted that the insertion of sodium‐ions is influenced not only by the availability of carboxylate groups, but also by the structural packing.

**Figure 4:**
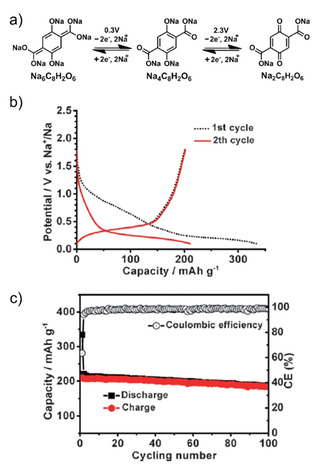
a) Chemical diagram showing redox reactions at two different voltages. b) The discharge/charge profile at initial cycles at current rate of 19 mAh g^−1^. c) Cycling performance over 100 cycles. Reproduced with permission.^47^ Copyright 2014, Wiley.

Thereafter, the concept of a full cell based entirely on organic electrodes was proposed by Chen and co‐workers.^47^ The electrochemical performance of tetrasodium dihydroxy‐2,5‐terephthalate was studied (Figure 4). The molecule bearing both phenolic enolate and carboxylate groups was found to exhibit a two‐step reversible redox at 2.3 V and 0.3 V, with insertion of 2 Na^+^ at both steps. The higher voltage process corresponded to the enolate groups, while the insertion of sodium ions to the carboxylate was observed at lower voltage. A specific energy of 65 Wh kg^−1^ at an average voltage of 1.8 V was reported for the full‐cell configuration. The cooperative electronic effect of the substituents was suggested to stabilize the redox states during cycling.

**Figure 5:**
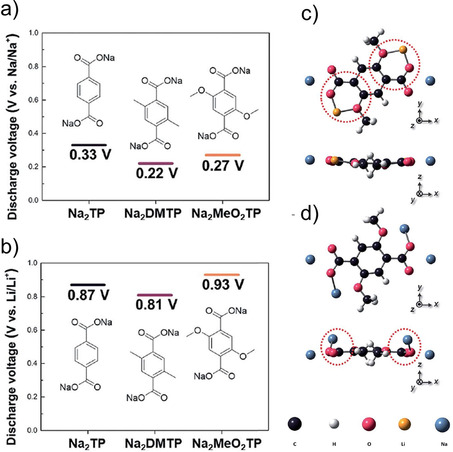
Discharge voltages for Na_2_TP and derivatives in a) Na cell and b) Li cell. Optimised molecular structures for Na_2_(MeO)_2_TP upon insertion of c) Li^+^ and d) Na^+^. Reproduced with permission.^49^ Copyright 2019, Royal Society of Chemistry.

Another example of an all‐organic sodium‐ion battery was reported by Shaijumon and co‐workers.^48^ A perylene based imide compound was used as the cathode while Na_2_TP served as the anode. Recently, the effect of substituents was examined in detail by Lee et al.^49^ A comparison of bare Na_2_TP with disodium compounds of terephthalates having electron donating groups viz. methyl (−CH_3_) and methoxy (−OCH_3_) was reported with cycling against both lithium and sodium‐ions. The discharge voltages were found to reduce in the case of cycling in Na‐ion cells for both linkers with pendant groups, as expected for electron‐donor groups (Figure 5). However, when the compounds were cycled in Li‐ion cells, the discharge voltage lowered only for methyl derivative, but rose for the methoxy substituted compound. DFT calculations suggested strong interactions for inserted Li^+^ with the oxygen atoms of the methoxy group, whereas steric crowding prevented such interactions in case of Na^+^. The authors noted that interactions between charge carrying ions and the substituents could override the electronic effect bestowed by the substituent. Another interesting study of substituents and the position of carboxylates was reported by Luo et al.^50^ A compound with two carboxylates at the ortho position of the phenyl ring (disodium phthalate – Na_2_PL) was examined. Another molecule with extra carboxylate substitution to the terephthalate (trisodium 1,2,4‐benzene tricarboxylate) was prepared. Na_2_PL did not show any activity, suggesting the importance of the position of active sites. On the other hand, the tricarboxylate compound exhibited reversible insertion of 2 Na^+^ ions and stable cycling performance was observed over 600 cycles, which was the result of the extra carboxylate group which reduced solubility in the electrolyte. Tripathi et al. reported a similar observation while investigating a symmetric tricarboxylate viz. trisodium‐1,3,5‐benzene tricarboxylate (Na_3_BTC).^51^ Detailed experimental studies and theoretical calculations were carried out on this compound. Although it has 3 carboxylate units, a reversible insertion of 1.8 Na^+^ was estimated. Unlike other carboxylate molecules, there is no reorganization of the π‐bonds, which suggests insertion solely by interaction with carbonyl oxygen atoms. This can be understood from the disfavored arrangement of π‐bonds during the loss of ring aromaticity upon conventional enolate formation.

Unlike pendant functional groups, Padhy et al. applied heteroatom doping within the 6‐membered aromatic ring.^52^ Disodium pyridine‐2,5‐dicarboxylate (Na_2_PDC) was prepared, which combines the effect of a heteroatom to improve conductivity and does not reduce the theoretical capacity of Na_2_TP. The compound showed reversible insertion of 2 Na^+^ per formula unit and rate performance of the compound was stable even at 5 C rate. Interestingly, unlike terephthalate compounds, Na_2_PDC exhibited two redox plateaus at 0.6 V and 0.4 V. Theoretical calculations and *ex situ* analysis hinted at insertion of Na^+^ at 0.6 V and interaction with the pyridyl N‐atom. Thereafter the expected insertion to the carboxylate is observed on the second plateau. Such N‐doping in the electrode mixture has been observed to improve electrochemical performance even in the case of LIBs.^53^

**Figure 6:**
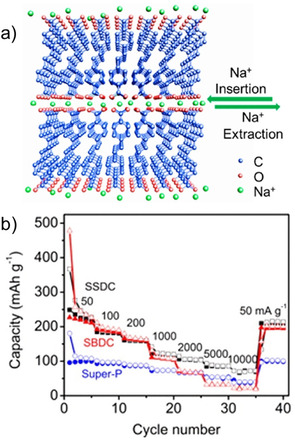
a) Figure showing the layered structure in Na_2_SDC (SSDC) for Na^+^ storage. b) Comparison of rate capability with Na_2_TP (SBDC). Reproduced with permission.^55^ Copyright 2015, American Chemical Society.

The extended version of terephthalate viz. 4,4′‐biphenyl dicarboxylate (BPDC) was tested as an anode material by Choi et al.^54^ Two sodiated forms of coordination polymers were synthesized, namely completely deprotonated (Na_2_BPDC) and partially deprotonated (NaHBPDC). A capacity of ∼200 mAh g^−1^ was reported for both the compounds, with more stable cycling for Na_2_BPDC over 150 cycles. *Ex situ* analysis suggested irreversible phase transformation for NaHBPDC, whereas the structure was retained over multiple cycles for Na_2_BPDC. A disodium compound of the extended version of BPDC − 4,4′‐stilbene‐dicarboxylate (SDC) was reported by Lei and co‐workers (Figure 6a).^55^ The presence of a carbon‐carbon double bond provided extra stability to the intermediate redox state by extended π‐electron conjugation. At a low current rate of 50 mA g^−1^, the compound delivered a stable capacity of ∼220 mAh g^−1^. Under similar testing conditions, the performance of Na_2_SDC was compared with Na_2_TP (Figure 6b). Although, Na_2_SDC has a significantly lower theoretical capacity than Na_2_TP, superior performance was noted both in terms of capacities and rate capability. At higher current densities of 1 A g^−1^, the cycling stability and Coulombic efficiencies over the initial cycles (80 % and 49 % respectively for the first cycle) were improved remarkably. This was ascribed to presence of π‐electrons which stabilize the redox states and strengthen molecular packing interactions. Previously, Mihali et al. had tested this approach of extending the π‐electron conjugation on disodium benzenediacrylate (Na_2_BDA).^56^ Although an initial Coulombic efficiency of 91 % was obtained, the cycling suffered a drastic decrease in specific capacity at all rates. The decomposition and dissolution of the material were cited as the reason for the absence of stability.

**Figure 7:**
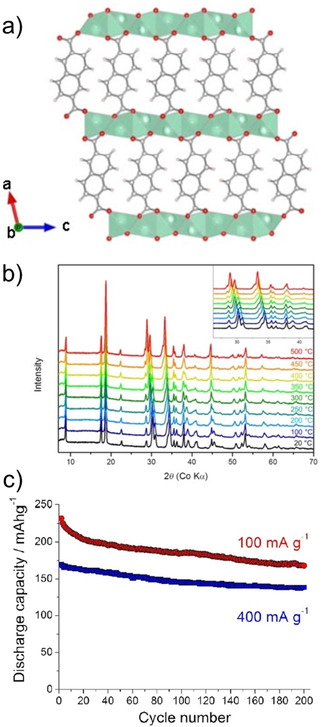
a) Structure of Na_2_NDC. b) High‐temperature PXRD (HT‐PXRD) patterns showing thermal stability. c) Cycling performance at 100 mA g^−1^ (red) and 400 mA g^−1^ (blue). Reproduced with permission.^59^ Copyright 2019, Wiley.

Another system which combines the extension of conjugated π‐electrons with theoretical insertion of 2 Na^+^ cations is disodium 2,6‐naphthalene dicarboxylate (Na_2_NDC). The naphthalene moiety comprises 10 π‐electrons and the rigid backbone can result in robust structures. An early example of using this molecule came from Deng et al.^57^ A 2‐step method was employed for the preparation of the electrode material. Instead of using the bare compound a nanocomposite with graphene was prepared. This composite facilitated better electronic conductivity and kinetics. The composite material exhibited stable cycling with 92 % retention of capacity after 100 cycles. Ramanujam and co‐workers reported cycling studies for the bare Na_2_NDC and examined a full cell with Na_3_V_2_O_2_(PO_4_)_2_F/rGO as the cathode.^58^ A two‐phase electrochemical process was observed with initial specific capacity of 208 mAh g^−1^. *Ex situ* studies suggested retention of the structure even after cycling. We reported a more detailed study of the compound which included structure elucidation, screening appropriate binder/carbon additive and *ex situ* analysis.^59^ The solid powders of Na_2_NDC were prepared by conventional acid‐base reaction. After detailed solid‐state characterization, the structure of the compound was elucidated from powder X‐ray diffraction (PXRD) patterns. The compound adopts a monoclinic structure, space group *P*2_1_/c, with π‐stacking between naphthalene rings and the organic layers separated by successive sodium layers (Figure 7a). The presence of non‐covalent interactions led to stabilization of the framework. This was reflected in the thermal stability of the compound, with structural integrity retained up to 500 °C (Figure 7b). Initial cycling studies were carried out using Super P and Kynar 2801 as the conducting carbon and binder. A two‐electron reversible redox in one step at a discharge potential of ∼0.4 V for the second cycle onwards was observed. However, to improve the cycling stability, another electrode composition containing Ketjenblack carbon and carboxymethyl cellulose (CMC) was tested. A much more stable cycling performance was observed with an initial capacity of 230 mAh g^−1^ (Figure 7c). Rate performance of this composition was stable and significant capacity of 133 mAh g^−1^ was reported for cycling at a rate of 5 C.

A more detailed study on multi‐ring aromatic systems was reported by Zhang and co‐workers.^60^ The authors chose 3,4,9,10‐perylene tetracarboxylic dianhydride (PTD) and its tetrasodiated form (Na_4_PTC). The central molecule was chosen with the motivation of extremely low solubilities of perylene derivatives and the presence of a planar conjugated π‐electron network. Upon cycling, PTD exhibited an initial charge capacity of 436.1 mAh g^−1^, with a stable capacity over 140 cycles. Detailed studies revealed the insertion of 4 Na^+^ ions without the opening of the anhydride ring, but rearrangement of π‐bonds. As a comparison, two anhydrides with fewer rings viz. 1,4,5,8‐naphthalene tetracarboxylic dianhydride and pyromellitic dianhydride were examined which showed similar capacities and redox potentials, suggesting a less significant role for the extent of π‐conjugation on Na^+^ storage. Likewise, cycling studies on Na_4_PTC also displayed transfer of 2 electrons from the ring with an initial charge capacity of 182.3 mAh g^−1^. The lower capacity suggested no specific effect of adding the extra Na^+^ ions to the PTD structure before cycling. Another comparison of ring systems without carboxylate groups viz. perylene, pyrene and truxene was performed. Although the 3 molecules have different structures and numbers of π‐electrons, all 3 presented low and similar discharge capacities. The authors inferred that there was no formation of Na/C complexes in planar multi‐ring aromatic molecules and suggested that for Na^+^ storage, the presence of active sites such as aromatic carbonyls was essential.

**Figure 8:**
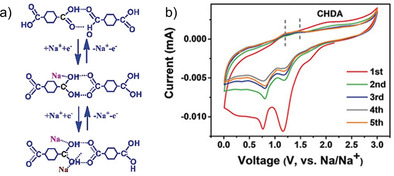
a) Proposed mechanism in CHDA for Na^+^ storage. b) Cyclic voltammetry profile for the first 5 cycles. Reproduced with permission.^61^ Copyright 2018, Wiley.

The weak influence of π‐electron rich systems for Na^+^ storage was also hinted by Ma et al.^61^ As in the above example, the direct solid form of a dicarboxylic acid viz. 1,4‐cyclohexanedicarboxylic acid (CHDA) was used as the electrode material. A high capacity of 284 mAh g^−1^ was observed, which was stable over 100 cycles. Unlike previously reported examples, theoretical calculations suggested the insertion of 2 Na^+^ by a completely different mechanism (Figure 8). Molecules without π‐bond backbones are held by strong H‐bonding interactions. During electrochemical reaction, there was a proton transfer between adjacent monomers, providing single bond character to both oxygen atoms of the carboxylate where two sodium‐ions are inserted. The authors inferred that the hydrogen transfer was the key step in the electrochemical response. The weak features of the anodic peaks were assigned to the weak electrode kinetics and hydrogen transfer reactions.

**Figure 9:**
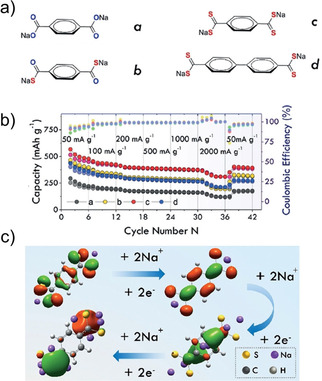
a) Molecules with different extent of S‐atom substitution. b) Rate capability for molecules a–d. c) Calculated HOMO energy levels and sites of interaction for Na^+^. Reproduced with permission.^63^ Copyright 2017, Wiley.

The role of careful molecular design was reiterated in a recent report by Wang and co‐workers.^62^ Disodium phthalate was earlier reported to be inactive for insertion of Na^+^ owing to loss of aromaticity. The authors joined 2 phthalate units via a carbonyl connection, thereby leaving room for π‐bond rearrangement during Na^+^ storage. Tetrasodium 5,5′‐carbonylbis(isobenzofuran‐1,3‐dione) showed the potential to accommodate 4 Na^+^ ions and exhibit a high capacity of 190 mAh g^−1^ with stable performance after 100 cycles. Unlike previous studies based on modulating properties by changing pendant linkages, Zhao et al. tuned the oxygen content of carboxylates by stepwise replacing it with sulfur (Figure 9).^63^ Initially two O‐atoms in Na_2_TP were replaced by two S‐atoms, and subsequently all four. The same tetra substitution was carried out for the longer version viz. Na_2_BPDC. While sulfur is expected to improve electronic conduction owing to higher electron density, the substitution led to a substantial increase in specific capacity also. The differences were also seen in operating potentials, which shifted to higher voltages. DFT calculations suggested that the presence of more electron dense S‐atoms led to the increase in the number of Na^+^ inserted. Wang and co‐workers observed a similar pattern for presence of S‐atoms.^64^ Disodium thiophene‐2,5‐dicarboxylate (Na_2_TDC) exhibited stable cycling with a high specific capacity of 334 mAh g^−1^. Theoretical calculations suggested the presence of S in the organic linker promoted extra insertion of Na^+^ ions and contributed to the improvement in electrical conductivity.

A few redox chemistries have been reported where the ion‐storage is concerned with the core of the molecule. Thereby, the participation of more sites in the compound improves the density of active sites and consequently capacities and cycling stability. Additionally, in systems where the carboxylate may not be available owing to steric hindrance or molecular packing interactions, such groups can still provide ion storage. The idea of using imine bond containing coordination compounds as redox active molecules was introduced and comprehensively studied by Castillo‐Martínez, Armand and co‐workers.65a The idea was based on the electrochemical process involving extended conjugation units (−COO‐phenyl‐C=N−).65b Several molecules were reported as control examples to study the effect of position and contribution from central phenyl moieties. It was observed that the molecular structure and atomic arrangements play a significant role in both the storage capacity and cycling stability. For the stabilization of long‐range conjugation, the out‐of‐plane configuration was inactive and the proximity of two N‐atoms also reduced the capacity on account of N−N repulsion. Wang and co‐workers extended this idea by examining the azo bond for sodium‐ion storage.^66^ In addition to studying the redox activity of azo bond, the authors reiterated the role of forming coordination bonded assemblies. Azobenzene was found to dissolve in organic electrolyte immediately and addition of one carboxylate did not improve the solubility issue. The compound with two carboxylate groups was stable to cycling. The positive effect of having redox active sites in the core was realized with the fast diffusion of Na^+^, leading to stable rate performance even at high current densities.

**Figure 10:**
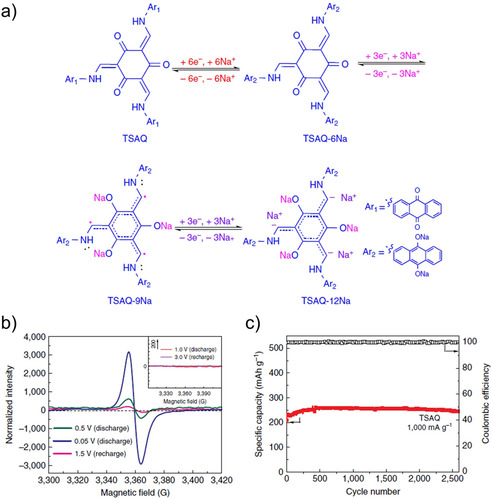
a) Proposed mechanism for Na^+^ insertion and stabilization of the radical. b) EPR profiles are different stages of cycling. c) Cycling performance of TSAQ (*tris* N‐salicylideneanthraquinoylamine) over 2500 cycles. Reproduced with permission.^67^ Copyright 2016, Nature Publishing Group.

The redox chemistry in carbonyl or imine bond systems involves formation of a carbon radical which is stabilized by rearrangement of unsaturated bonds. Wu et al. reported an interesting example wherein the carbon radical was stabilized by the local environment and could be used to reversibly insert about 0.83 Na^+^ ions per radical site (Figure 10).^67^ This led to both higher capacity and prolonged cycling stability even at higher rates. The radical stabilization was realized by the resonance effect of the connected −NH−Ar groups and the steric hindrance caused by the rigid structure.

#### 3.1.2 Potassium coordination compounds

Ketones usually show redox at higher voltages, making them suitable as cathode materials. However, an early study of organic anodes was reported on oxocarbon viz. M_2_(CO)_*n*_ (where M=Li, Na, K and *n*=4, 5, 6) by Chen and co‐workers.^68^ The authors reported that the 4 membered rings did not show any significant capacity, but 5 and 6‐membered systems did show moderate to high capacities. The potassium compound (K_2_C_6_O_6_) had two reversible peaks making it a useful anode and cathode material, with the possibility of inserting 2 K^+^ ions. The studies also revealed that K_2_C_6_O_6_ exhibits semiconductor behavior, requiring negligible carbon additive. Following the development of NIB anodes, dipotassium terephthalate has been a molecule of significant interest in KIBs as well. Initial studies came from Lei et al.,^69^ and Deng et al.^70^ The former group presented cycling studies of K_2_TP and reported a capacity of 229 mAh g^−1^ using DME as the electrolyte solvent. The electrolyte was chosen to provide a stable SEI. Additionally the authors studied a full organic cell with K_2_(CO)_6_ as the cathode. The full cell configuration was found to exhibit an energy density of 52 Wh kg^−1^. The latter group extended the study to include the pyridine analogue viz. K_2_PDC. At low current densities (0.05 C), high capacities of 270 and 245 mAh g^−1^ were observed for K_2_TP and K_2_PDC. However, at high current rates, K_2_PDC exhibited better performance, which was ascribed to a heteroatom induced change in HOMO‐LUMO energies. The potassium compound of the regioisomer with N‐atom inclusion was recently reported by Li et al.^71^ The N‐atom was found to influence the ion insertion and a 3‐step redox was observed. Wang et al. examined the effect of graphene oxide wrapping on K_2_TP.^72^ An improved capacity of 212 mAh g^−1^ at a rate of 200 mA g^−1^ over the bulk material was noted with stable cycling performance after 400 cycles. The wrapping of graphene oxide was believed to contribute to better electronic conductivity, and retention of structural stability during cycling. Fan and co‐workers explored the performance of terephthalic acid (H_2_TP) as a negative electrode for all three of Li/Na/K‐ions.^73^ Average capacities were found to be 235 mAh g^−1^ (500 cycles), 200 mAh g^−1^ (50 cycles) and 240 mAh g^−1^ (150 cycles) respectively. Although the electrochemical performance was interesting, organic carboxylic acids liberate H_2_ gas at low redox potentials owing to conversion of protons, and hence the salt forms are more useful. However, the stable cycling for all 3 ions emphasized the value of organic materials for application to batteries having similar electrochemistry. The extended dicarboxylate versions of biphenyl (BPDC) and stilbene (SDC) were studied in detail by Li et al.^74^ Both were found to have better conductivities and stable cycling. The potassiation voltages were subtly different 0.35 V for K_2_BPDC, compared to 0.55 V for K_2_SDC. The effect of π‐conjugation was seen in the performance at high current rate of 1 A g^−1^, where K_2_SDC showed an average capacity of 75 mAh g^−1^ for 3000 cycles.

The utilization of extended multi‐ring aromatic systems has been studied using different molecular backbones. Li et al. reported the two‐electron redox activity of potassium naphthalene‐2,6‐dicarboxylate (K_2_NDC).^75^ A low redox potential of ∼0.55 V was reported with a capacity of 139 mAh g^−1^ at a current density of 100 mA g^−1^. The composite with carbon nanotube was found to improve the kinetics of ion insertion and reduce polarization.

**Figure 11:**
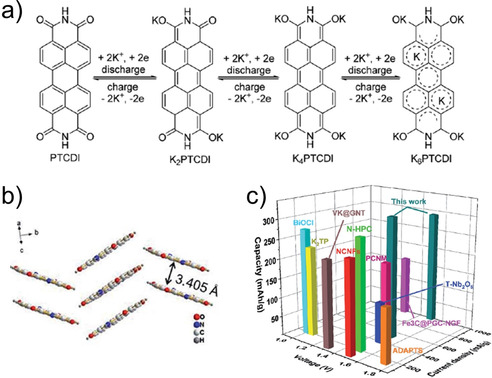
a) Proposed mechanism for K^+^ storage in perylene based diimide system. b) Structural packing showing presence of stacking interactions. c) Comparison of performance with reported anode materials for KIB. Reproduced with permission.^78^ Copyright 2019, Royal Society of Chemistry.

An early example of utilizing perylene core as electrode material was reported by Hu and co‐workers.^76^ The authors investigated electrochemical performance of 3,4,9,10‐perylene‐tetracarboyxlic dianhydride (PTCDA), which was found to useful as both cathode and anode with multi‐step K^+^ insertion. The organic molecule could insert up to 11 K^+^ cations when discharged to 0.01 V. Fan and co‐workers studied tetrapotassium 3,4,9,10‐perylene tetracarboxylate (K_4_PTC), which leads to better electronic conduction.^77^ The lower density of active sites reduces the theoretical capacity <100 mAh g^−1^ for 2 K^+^ ion storage, as electronic rearrangement forbids 4 ion inclusion. However, the robust structure after preparing a composite with carbon nanotubes (CNT) delivered a stable rate performance, even at higher current densities of 0.5 A g^−1^. Bai et al. presented an interesting approach to circumvent the limited capacity of K_4_PTC by using an imide viz. 3,4,9,10‐perylenetetracarboxylic diimide (PTCI).^78^ The organic molecule with poor solubility exhibited very low resistance to charge‐transfer and delivered an average capacity of 310 mAh g^−1^ at current density of 0.5 A g^−1^. DFT calculations suggested that up to 6 K^+^ ions could be inserted in the molecule, with 4 at the enolate position and the additional 2 binding to the delocalized π‐electrons in the rings (Figure 11). The use of biomolecules with carbonyl functional sites was reported by Xue et al.^79^ The authors examined Vitamin K3 (2‐methyl‐1,4,–naphthoquinone) by forming a composite with graphene nanotubes (GNT). The composite delivered high discharge capacities of 165 mAh g^−1^ at a current rate of 1 A g^−1^. *Ex situ* analysis suggested insertion of K^+^ at both the available carbonyl positions.

**Figure 12:**
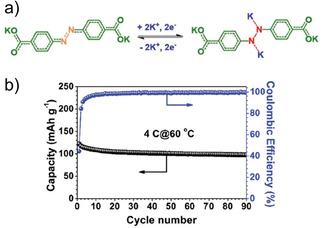
a) Proposed mechanism for K^+^ insertion in azo based molecule. b) Cycling performance at high current rate and elevated temperature (60 °C). Reproduced with permission.^80^ Copyright 2019, Wiley.

The use of the azo bond for K^+^ storage was proposed by Wang and co‐workers (Figure 12).^80^ The dipotassium compound of azobenzene‐4,4′‐dicarboxylate was cycled against K^+^ and was found to exhibit reversible insertion of ∼2 ions. Apart from stable cycling, the compound was found to perform well even at higher temperatures of 50 °C and 60 °C.

### 3.2 Porous materials

Organic building blocks are employed for the conventional organic polymers and the recently emerging porous materials – metal‐organic frameworks (MOFs) and porous organic polymers (POPs), including covalent organic frameworks (COFs). Conventional organic polymers based on unsaturated bonds as anodes for ion storage have long been reported,^81^ including naturally derived polymers^82^ or assembly in full organic cells,^83^ but typically are useful as cathode materials.^84^ Crystalline networked solids such as MOFs/COFs have recently emerged as functional solid‐state materials with several promising features.^85^ Some of the recent results on Na^+^/K^+^ ion storage based on porous solids are discussed below. These ordered crystalline solids are also attractive candidates for obtaining nanostructured carbons or composites, and hence usually the derivatives are employed for battery applications.^86^ Also in certain cases during cycling the pristine phases undergo conversion into amorphous carbonaceous materials which have ion storage properties.^87^

#### 3.2.1 NIB anode materials

**Figure 13:**
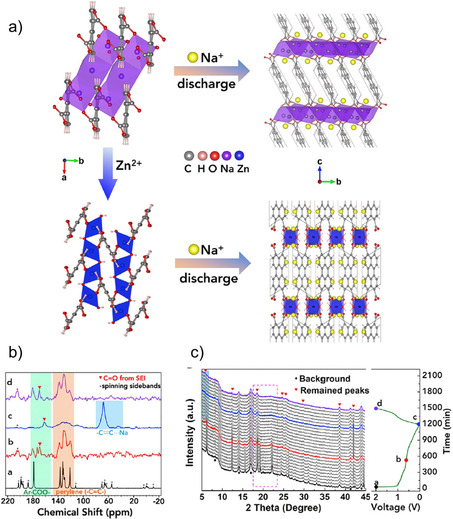
a) Packing diagram for Zn−PTC and Na_4_−PTC and corresponding Na^+^ ion insertions. b) Solid‐state NMR and c) *in‐situ* PXRD patterns at different stage of cycling showing activation of unsaturated bonds and structural stability. Reproduced with permission.^89^ Copyright 2018, Elsevier.

An example of a MOF based anode was reported by Wang and co‐workers.^88^ A Co^II^ MOF having a BPDC linker was prepared and investigated for insertion of Na^+^ ions. A high capacity with remarkable cycling stability was reported. The length of the linker directing the porous nature of the structure for smoother ion insertion/extraction was attributed as the dominant process. Liu et al. also reported an example highlighting the efficacy of MOFs.^89^ The authors prepared a Zn^II^‐based MOF constructed from the perylene tetracarboxylic acid ligand (Figure 13). The compound delivered a high specific capacity with significant stability. Unlike the sodium analog (Na_4_PTC),^60^ the authors observed that the MOF could activate the perylene ring and up to 8 ions could be inserted. This was attributed to the presence of a wavy open framework and local structure arrangement.

Significant efforts have been devoted to improving the electrical conductivity of MOFs. While several noteworthy compounds and approaches are reported, these materials generally do not exhibit redox features. Bao and co‐workers reported an example wherein a conductive Co^II^‐based MOF exhibited reversible insertion of Na^+^ ions.^90^ The compound comprising hexaaminobenzene (HAB) linkers had a bulk conductivity of 1.57 S cm^−1^. A redox reaction comprising a transition from a quinoid imine in Co‐HAB to a benzoid amine led to a high specific capacity of 291 mAh g^−1^ along with stable cycling. The authors also reported that the MOF had significantly higher areal capacities than well studied anode materials. Wang and co‐workers reported an example of a Ni^II^‐based conjugated coordination polymer, synthesized from a tetramine linker 1,2,4,5‐benzenetetramine (BTA).^91^ A 3‐electron storage mechanism was observed, involving transformation of the −C=N bond to −C−N single bonds along with reduction of Ni^II^ to Ni^I^. The formation of Ni^I^ was confirmed by catalytic activity of the redox state. The compound Ni−BTA was found to deliver a high capacity of 500 mAh g^−1^ at a current density of 0.1 A g^−1^.

A popular choice of preparing COFs involves formation of imine bonds. A material with extensive and homogenous distribution of such redox active sites is highly suited to ion storage. Patra et al. demonstrated the utility of COFs constructed from imine bonds for Na^+^ ion insertion and extraction.^92^ The porous organic solid having extensive π‐bond conjugation with alternate triazine moieties exhibited low operating voltage, good capacity retention and stable rate performance. The insertion was ascribed to interaction of Na^+^ with both the imine and triazine N‐atoms. Likewise, a porous polymer built from azo linkages was studied for Na^+^ ion storage by El‐Kaderi and co‐workers.^93^ Reversible insertion of 4 Na^+^ per repeating unit was reported. However, the working potential was significantly higher (>1 V).

**Figure 14:**
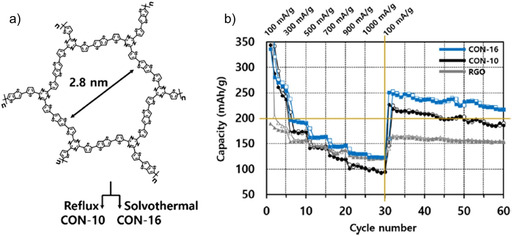
a) Molecular structure of CON‐10 and CON‐16. b) Rate performance for the two compounds relative to RGO (reduced graphene oxide). Reproduced with permission.^94^ Copyright 2018, American Chemical Society.

Kim and co‐workers observed effective Na^+^ storage in covalent organic nanosheets (CONs) prepared by Stille cross‐coupling in a series of compounds with varying pore sizes (Figure 14).^94^ The compound with higher densities of N and S‐atoms was reported to have superior capacities and cycling stability. The charge storage was linked to the interaction between S‐functional sites and Na^+^. Also, the ion transport features were seen to be linked to the planarity of the structure and conformational flexibility. Recently Yang et al. also investigated extended S‐rich conjugated microporous polymers for ion storage.^95^ The polymer with higher branching was found to present superior insertion of both Li^+^ and Na^+^ ions. This observation was linked to varying electronic features of the two porous molecules.

#### 3.2.2 KIB anode materials

In principle, porous materials present the ability to accommodate larger size K^+^ ions without structural distortion and provide channels for smooth ion diffusion. However, this aspect of research is relatively young and only a few examples have been reported. Feng and co‐workers tested K^+^ storage in the highly robust MOF, MIL‐125(Ti).^96^ The MOF built from terephthalate (TP) has a surface area of ∼1100 m^2^ g^−1^ and a pore diameter of 1.60 nm. The compound exhibited remarkably stable cycling and *ex situ* studies confirmed the reversible insertion of K^+^ to the organic carboxylate ligand. A similar observation was reported by Deng et al. using the terephthalate based MOF (MOF‐235) having Fe^III^ metal nodes.^97^ Instead of using the bare MOF, its composite with multiwall carbon nanotubes (MWCT) was prepared *in situ*. The *ex situ* analysis during cycling suggested that K^+^ inserted into the carboxylate moiety of the compound.

**Figure 15:**
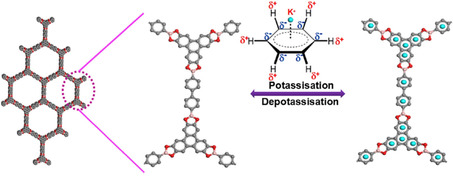
Packing diagram of COF‐10 and the predicted interaction of K^+^ with the π‐rich core of the compound. Reproduced with permission.^98^ Copyright 2019, American Chemical Society.

Previously Wang and co‐workers had reported a similar strategy of growing a boronic ester based COF over carbon nanotubes (CNT) and studied its ability to store K^+^ ions (Figure 15).^98^ The composite was able to provide fast and smooth K^+^ kinetics and exhibited cycling stability over 4000 cycles. The interaction of K^+^ with a π‐rich triphenylene core (cation‐π) was found to be the underlying cause for K^+^ storage.

## 4 Conclusion and Outlook

Sodium‐ion (NIB) and potassium‐ion batteries (KIB) are likely to be an integral part of next‐generation energy storage devices. The battery materials used for commercially successful Li‐ion batteries (LIBs) cannot be directly employed for NIBs or KIBs. In particular, on the anode side there is immense interest in the development of suitable materials. Organic redox‐active molecules having low‐voltage electrochemistry are well suited for this purpose. In addition, the inherent benefits of environmentally sustainable, structurally diverse and cost‐effective materials have further propelled research. Among the different active moieties for the anode, carboxylate, imine and azo bonds have been exploited with reasonable success for reversible insertion of Na^+^ and K^+^ ions. The materials based on such molecules have demonstrated potential with moderate‐to‐high capacities at low but safe voltages. However, some optimization is required in terms of improving cycling stabilities, low electronic conductivity, and kinetics for these materials to compete with frontrunners in this area of research.

The access to structure‐property correlation permits organic materials to exploit molecular engineering at multiple levels. The quest for unexplored redox‐active organic groups continues to drive fundamental research. The early success of advanced porous materials like metal‐organic frameworks (MOFs) and covalent organic frameworks (COFs), towards stable and fast cycling of heavier ions (Na^+^/K^+^) is likely to propel development of new materials or approaches. In particular, the stability of such solids in organic solvents may provide a robust pathway to harness the features of organic redox into stable electrodes. Apart from efforts in molecular engineering to improve electrochemical parameters, methods to prepare scalable materials is another aspect of upcoming research. The widening scope of rechargeable battery technology will require sustainable solutions for future energy demands. Organic materials which have exhibited limited success thus far are likely to feature more substantially in this regard. The research on such redox‐active molecules is young and will require comprehensive evaluation of different classes of compounds and materials to fully realize its potential.

## Conflict of interest

The authors declare no conflict of interest.

## Biographical Information

*Dr. Aamod V. Desai completed an integrated MS‐PhD from Indian Institute of Science Education and Research (IISER) Pune, India in 2018. His research was focused on ion‐exchange applications of metal‐organic frameworks (MOFs). He joined the University of St Andrews in 2019 as a postdoctoral research fellow and is currently working on coordination polymers for rechargeable batteries*.

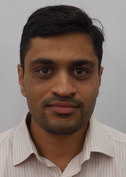

## Biographical Information

*Prof. Russell E. Morris completed his education from University of Oxford where he obtained BA and DPhil. He is currently Bishop Wardlaw Professor of Chemistry at University of St Andrews. His research interest lies in addressing challenges in synthesis and applications of porous materials such as zeolites and metal‐organic frameworks (MOFs). He is a Fellow of the Royal Society (FRS), a Fellow of the Royal Society of Edinburgh (FRSE) and a Fellow of the Learned Society of Wales (FLSW). He is the Chair of the Editorial Board of Dalton Transactions*.

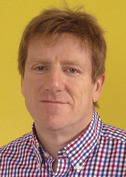

## Biographical Information

*Dr. A. Robert Armstrong is a senior researcher at the University of St Andrews. His research is concerned with the synthesis and characterization of novel electrode materials for rechargeable batteries, with emphasis on correlating structure and properties using powder X‐ray and neutron diffraction. He is joint project leader on the Faraday Institution sodium‐ion battery project NEXGENNA*.